# AI-Driven Framework for Recognition of Guava Plant Diseases through Machine Learning from DSLR Camera Sensor Based High Resolution Imagery

**DOI:** 10.3390/s21113830

**Published:** 2021-06-01

**Authors:** Ahmad Almadhor, Hafiz Tayyab Rauf, Muhammad Ikram Ullah Lali, Robertas Damaševičius, Bader Alouffi, Abdullah Alharbi

**Affiliations:** 1 Department of Computer Engineering, Networks Jouf University, Sakaka 72388, Saudi Arabia; aaalmadhor@ju.edu.sa; 2 Independent Researcher, Bradford BD8 0HS, UK; 3 Department of Information Sciences, University of Education Lahore, Lahore 41000, Pakistan; m.i.lali@ue.edu.pk; 4 Faculty of Applied Mathematics, Silesian University of Technology, 44-100 Gliwice, Poland; robertas.damasevicius@polsl.pl; 5 Department of Computer Science, College of Computers and Information Technology, Taif University, P.O.Box 11099, Taif 21944, Saudi Arabia; balouffi@tu.edu.sa; 6 Department of Information Technology, College of Computers and Information Technology, Taif University, P.O. Box 11099, Taif 21944, Saudi Arabia; amharbi@tu.edu.sa

**Keywords:** guava fruit diseases, feature extraction, machine learning, agricultural informatics

## Abstract

Plant diseases can cause a considerable reduction in the quality and number of agricultural products. Guava, well known to be the tropics’ apple, is one significant fruit cultivated in tropical regions. It is attacked by 177 pathogens, including 167 fungal and others such as bacterial, algal, and nematodes. In addition, postharvest diseases may cause crucial production loss. Due to minor variations in various guava disease symptoms, an expert opinion is required for disease analysis. Improper diagnosis may cause economic losses to farmers’ improper use of pesticides. Automatic detection of diseases in plants once they emerge on the plants’ leaves and fruit is required to maintain high crop fields. In this paper, an artificial intelligence (AI) driven framework is presented to detect and classify the most common guava plant diseases. The proposed framework employs the Δ E color difference image segmentation to segregate the areas infected by the disease. Furthermore, color (RGB, HSV) histogram and textural (LBP) features are applied to extract rich, informative feature vectors. The combination of color and textural features are used to identify and attain similar outcomes compared to individual channels, while disease recognition is performed by employing advanced machine-learning classifiers (Fine KNN, Complex Tree, Boosted Tree, Bagged Tree, Cubic SVM). The proposed framework is evaluated on a high-resolution (18 MP) image dataset of guava leaves and fruit. The best recognition results were obtained by Bagged Tree classifier on a set of RGB, HSV, and LBP features (99% accuracy in recognizing four guava fruit diseases (Canker, Mummification, Dot, and Rust) against healthy fruit). The proposed framework may help the farmers to avoid possible production loss by taking early precautions.

## 1. Introduction

Guava is grown in tropical and subtropical climates that are conducive to its development [1]. It is high in calcium, vitamin C, nicotinic acid, phosphorus, and soluble fiber, providing an important source of food for many less developed countries. Guava is thought to have originated in South America (Mexico to Peru) [2]. South Asian nations, the Hawaiian Islands, Cuba, Brazil, Pakistan, and India produce most of it.

Mummification, guava wilt, fruit canker, fruit spot, Stem canker, Leaf blight, Rust of guava, Fruit rot, and dry rot are severe diseases that can reduce the overall yield of guava plants. Anthracnose [2], which was first discovered by Mehta in Uttar Pradesh in 1951, is a disease that affects guava plants, the first study of guava wilt was made by Gupta [3], fruit canker was first reported from Bombay [4] caused by Pestalotia psidii Pat, fruit spot caused by alga was first reported by Ruehle [5] and, Guava stem canker was first discovered in Patharchatta [6]. Mitra (1929) reported Dastur, and in 1969 at Vellayani, 40% of the fruit were infected with dry rot [7]. To control these type of diseases, different types of fungicides and chemicals applied on guava crop, but it affects environment badly and cause economic loss.

Pakistan’s main natural resources are agricultural land and water. 25% of agriculture accounts for about 25% of Pakistan’s GDP and hires about 43% of the workforce [8]. In a country’s economic growth, the agricultural sector is critical. The fact that agriculture growth is primarily responsible for Pakistan’s GDP growth has been reported and sponsored. If the agricultural sector’s growth rate is poor, the country will face a shortage of food and other essential raw materials. Plants provide food and are essential sources of energy-rich compounds, vitamins, and minerals; in Pakistan, the Guava is a common fruit. Pakistan is the world’s fourth-largest guava producer. This fruit was produced in Pakistan in 1,784,300 metric tons [9].

In this case, the most important thing is to make an accurate and timely diagnosis of the disease. If these diseases are not correctly identified and managed, they will have a negative impact on the next generation of guavas. Close observation is required by managing intermittent yields to check which disease significantly impacts crop production after harvest time. Experts can identify and classify guava disease based on symptoms. This, however, necessarily requires manual observation and continuous testing, which can be error-prone and costly. A large portion of the agriculturists in underdeveloped countries are uneducated and unaware of non-native diseases. As a result, the former will have to drive long distances to find a well-trained specialist, which could be time-consuming and costly [10]. As a result, plant diseases are a severe hurdle towards achieving agricultural sustainability in developing countries [11,12].

One way to address this problem is to use computer technology for the detection of plant diseases. Such a system would either substitute experts or have a second opinion on an expert’s decision. As this solution is cost-effective and straightforward [13], the farmers can take corrective measures to avoid the disease’s spread [14]. Researchers have developed various diagnostic systems with the help of computer technology to diagnose various crop diseases [14]. These systems feed by RGB images that are red, blue, and green as input then decide either image is healthy or unhealthy if the image fed as input ( $I_{INPUT}$ ) is unhealthy. Segmentation applied separates them into standard image ( $I_{normal}$ ) and diseased image ( $I_{diseased}$ ) as in Equation (1) and feature extraction technique is applied to the diseased part for classification.

(1) $$I_{INPUT}=I_{normal}+I_{diseased}$$

The contribution of the research is as follows:

- A Guava disease classification framework based on guava plant images is proposed. The proposed framework separates the Guava images into the diseased image ( $I_{D}$ ) and non-diseased ( $I_{ND}$ ) image. The proposed approach’s primary goal is to detect the disease present in guava plant images.
- Image-level and disease-level-based feature extraction approaches are used to obtain robust guava disease recognition.
- The corresponding disease-segmented image with a specific label is assigned a class, which gives information about the disease. Four guava diseases, such as Canker, Mummification, Dot, Rust, and one extra target class, “healthy”, are covered in the presented study.
- The proposed framework is evaluated on a high-resolution image dataset.

The high-resolution images cannot be handled using conventional deep convolutional neural network architectures without significant reduction of the resolution, so a large part of the information contained in images is lost, which affects the performance of image segmentation, and classification negatively [15,16]. Therefore, we adopted a combination of computer-vision and machine-learning techniques.

The remaining paper is organized as follows. Recent related work with the summary of existing methodologies is given in Section 2. A proposed method including data pre-processing, feature extraction approaches, segmentation, and classification are presented in Section 3. Section 4 contains the dataset description, experimental results, and analysis. The study is concluded in Section 5.

## 2. Related Work

Previously, most researchers relied on image processing, pattern classification, and machine-learning techniques, especially in agriculture [17,18,19]. Video cameras are used to capture images from the environment first. Then, to extract useful features from images, some operations performed on the image [20]. The detection of diseased regions in an image is the core objective. There has been an increasing growth of research focusing on plant disease classification in recent years aiming to develop effective plant diagnostics systems for farmers [21,22,23]. A variety of Artificial Intelligence (AI) methods have been adopted in classifying and detection various plant diseases such as olive [24], pomegranate [25], plum [26], rice [27], tomato [28], cassava [29], mango [30], tea leaf [31], apple [32], citrus [33], oranges [34], etc.

For the diagnosis of plant diseases, various methods have been presented. Some of the promising techniques to diagnose the disease in plants are discussed below. In [35] presented a tool for detecting citrus diseases that can be done automatically. They use the algorithm that was denoted as Δ E algorithm that uses the color difference to define the region affected by the disease and the color histogram and textural features used for classification purposes.

The authors in [36] developed a multi-spectral camera system that can detect defects on citrus surfaces in real time by capturing visual and close proximity images from the same scene. In [37], the author proposed novel segmentation techniques to segment the lesion areas affected by anthracnose. Standard and anthracnose effects on fruit were categorized using neural network (NN) classifiers. In [38], texture characteristics are used to identify plant leaf diseases, and a technique to detect unhealthy regions of plant leaves has been suggested. For the segmentation of leaf decay ailment disease in betel vine leaf image-processing and computer-vision algorithms proposed by Dey et al. in [39]; threshold known as Otsu was applied. In [40] cellular automate filter is used to process the input leaf images. For detecting a disease named bacterial blight, which is present in pomegranate fruitlet, an image-processing approach is proposed in [41]. At an early stage, corn/weed conditions were identified in [42] using Back Propagation Neural Network. Pydipati et al. in [43], create a color matrix CCM as a function of texture, using the adverse influence of images in the HSI color space. Moreover, Napoli et al. [44] suggested using the simplified firefly algorithm to search for critical areas in images for recognizing the target areas of interest.

Zhang and Chaisattapagon suggested a color-based weed detection method for Kansas wheat [45].

In [46], color indices were created to distinguish weeds in various environments, including dirt, rubble, and lightening. An algorithm based on statistics collected from local maxima and minima was proposed in [47] to extract leaf/plant shape features.

Crowe and Delwiche [48] used two combined near-infrared (NIR) images of fruit to create an algorithm for analyzing apple and peach defects. Edwards et al. [49] used various pattern recognition models to distinguish surface blemishes on different apple varieties, including multi-layer backpropagation, KNN, and nearest cluster algorithms are both unimodal Gaussian algorithms. They used reflectance spectra of the whole tree to assess the damage caused by citrus blight disease on citrus plants.

An innovative algorithm for lesion area extraction is presented in [50]. To recognize and classify the type of disease, first-order statistical features based on texture are extracted from the lesion region. Objects are then categorized based on their texture characteristics. The authors in [51] describes a legal remote sensing technique for monitoring plant diseases in arable crops at an early stage of disease production from the ground. Khamparia et al. [52] adopted a hybrid method for recognizing crop leaf diseases using the combination of convolutional neural networks (CNNs) and autoencoders.

The related works are summarized in Table 1.

Critics (advantages and drawbacks) of the related works are presented in Table 2.

## 3. Methodology

**Figure 1:**
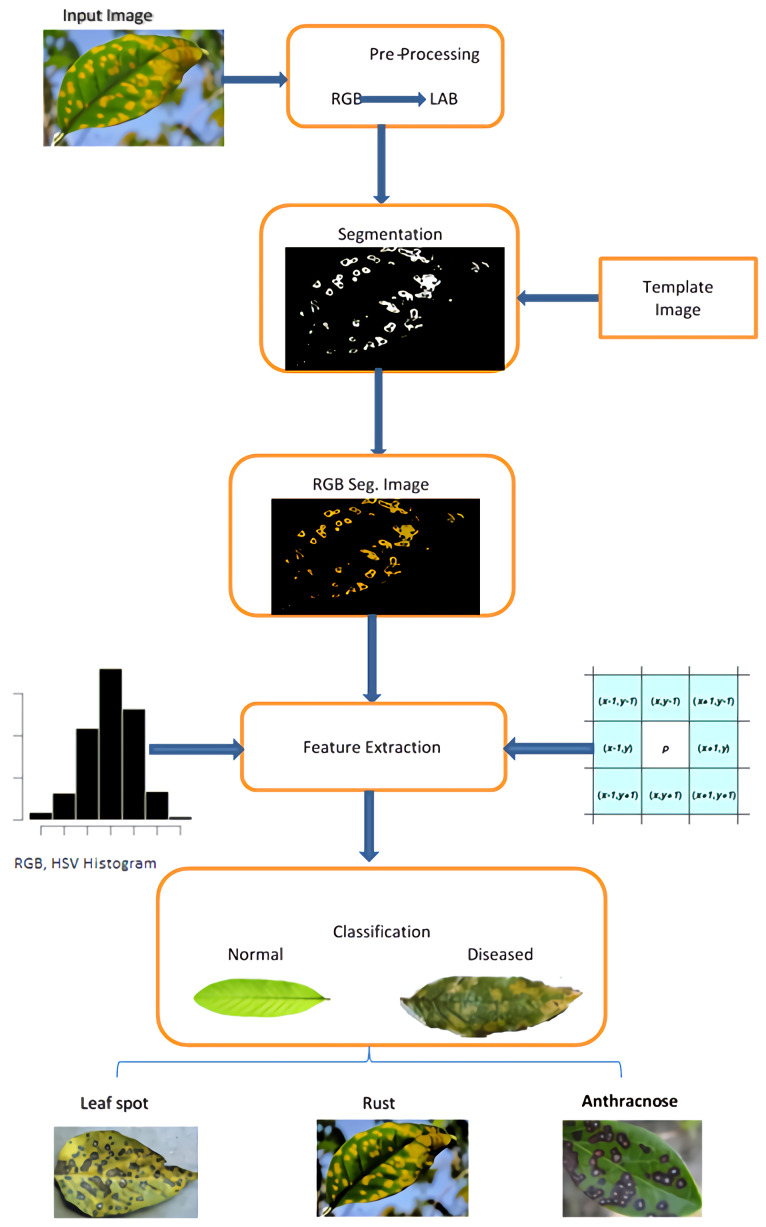
Workflow of Guava disease classification system.

The presented framework uses a computer-vision-based approach to classify guava plants’ leaves and fruit in diseased and non-diseased images. Furthermore, it assigns a diseased image to a particular disease group. Figure 1 depicts the proposed system’s workflow diagram. The following are the stages of our suggested procedure:

1. Pre-processing of image.
2. Segmentation of image.
3. Feature extraction.
4. Classification.

### 3.1. Image Pre-Processing

Researchers used a digital camera to capture guava leaves from various orchards to improve the sample’s uniformity and obtain more accurate image data. Image acquisition plays a significant role in this system. Even if the image were improved, the desired results could not be produced if the image were not captured properly. The obtained images are then resized to 256×256 pixels size. Image pre-processing techniques enhance the features, reduce distortions, and make the image more compelling. The lowest abstraction stage is color space conversion, and image processing [64].

### 3.2. Image Enhancement

To improve the quality of digitally stored images, various image-processing methods are used. One improved distribution’s values are mapped to the values of another improved distribution. Histogram equalization is used to improve the contrast of the transformed input image. Because images are captured in various lighting conditions, some images contain bright regions while others contain dark regions, resulting in an unbalanced histogram. The enhanced image is then normalized. The Normalization perform in Equation (2).

(2) $${H(p}_{\left(x,y\right)}=Round\left(\frac{f_{cdf}P_{\left(x,y\right)}-f_{{cdf}_{min}}}{\left(R\times C\right)-f_{cdf}}\times L-1\right)$$

where $f_{cdf}\;$ is the gray level cumulative frequency, and $f_{{cdf}_{min}}$ represents minimum value of cumulative distribution function. The R × C stands for the total number of pixels in each row and column, and the L stands for the total number of intensities. $f_{cdf}P_{\left(x,y\right)}$ is the current pixel’s intensity.

### 3.3. Color Space Transformation

The RGB color range is not advised for color-based detection and color assessment due to the nonuniform features and mixing of chrominance and luminance information [65]. Sometimes some desired information remains invisible in RGB color space; then, color space transformation is applied to those images to acquire explicit information. The proposed algorithm includes the image’s L*, a*, and b* component values. Consequently, the image fed into the device as input must be translated from RGB to LAB color space. First, RGB to XYZ conversion. In the human color vision, the XYZ color space perceives colors [53]. CIE (International Commission on Illumination) created the XYZ color space in 1931 while conducting experiments on human perception [46]. Equation (3) shows how to transform the RGB color space to the XYZ color space using a matrix.

(3) $$\left[\begin{array}{c} X\\ Y\\ Z \end{array}\right]=\left[\begin{array}{ccc} 0.412453 & 0.357580 & 0.180423\\ 0.212671\; & 0.715160\; & 0.072169\\ 0.019334 & 0.119193 & 0.950227 \end{array}\right]\times \left[\begin{array}{c} R\\ G\\ B \end{array}\right]$$

Furthermore, XYZ to LAB color space conversion is performed as in Equations (4)–(10).

(4) $$X^{\prime }=\frac{X}{95.047},Y^{\prime }=\frac{Y}{100.00},Z^{\prime }=\frac{Z}{108.883}$$

(5) $$X^{\prime }=\left\{\begin{array}{cc} \;\;\;\;\;{\left(X^{\prime }\right)}^{1/3} & \;\;\;\;\;\;\;\;\;\;\;\;\;forX^{\prime }>0.008856\\ \left(7.787\times X^{\prime }\right)+\frac{16}{116} & \;\;\;\;\;\;\;\;\;\;\;\;\;Otherwise \end{array}\right.$$

(6) $$Y^{\prime }=\left\{\begin{array}{cc} \;\;\;\;\;{\left(Y^{\prime }\right)}^{1/3} & \;\;\;\;\;\;\;\;\;\;\;\;\;for\;Y^{\prime }>0.008856\\ \left(7.787\times Y^{\prime }\right)+\frac{16}{116} & \;\;\;\;\;\;\;\;\;\;\;\;\;Otherwise \end{array}\right.$$

(7) $$Z^{\prime }=\left\{\begin{array}{cc} \;\;\;\;\;{\left(Z^{\prime }\right)}^{1/3} & \;\;\;\;\;\;\;\;\;\;\;\;\;for\;Z^{\prime }>0.008856\\ \left(7.787\times Z^{\prime }\right)+\frac{16}{116} & \;\;\;\;\;\;\;\;\;\;\;\;\;Otherwise \end{array}\right.$$

(8) $$L=(116\times Y^{\prime })+16$$

(9) $$a=500\times (X^{\prime }-Y^{\prime })$$

(10) $$b=200\times (Y^{\prime }-Z^{\prime })$$

### 3.4. Image Segmentation

**Figure 2:**
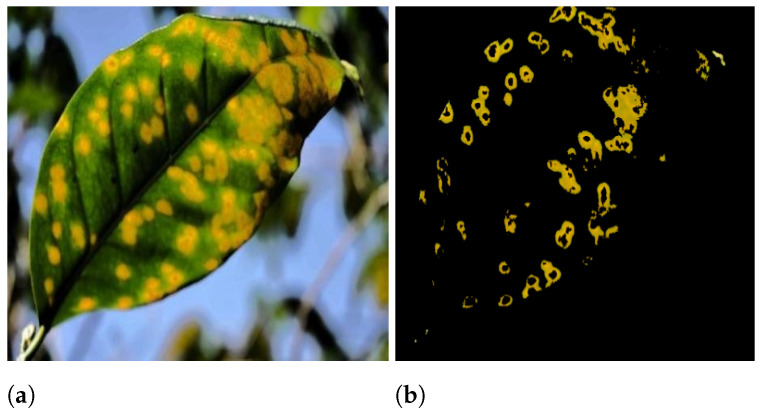
(**a**) leaf affected with guava rust (input image), (**b**) diseased area.

In many practical applications, including object recognition, computer vision, and medical image analysis [45], segmentation is one of the most important and challenging problems. Image segmentation is the process of dividing an image into several parts. Segmentation aims to make an image’s representation more relevant and intuitive by simplifying it. Figure 2a shows the input image and Figure 2b show the diseased part. After conducting segmentation, the diseased region is subjected to feature extraction. The image segmentation process is divided into two steps.

#### 3.4.1. Delta E ( Δ E)

We used an algorithm known as Δ E to segment the image by calculating the distance between the colors in the LAB color space [66]. This algorithm saves a prototype of an image’s symptom before segmenting the enhanced image using tolerance and the enhanced image energy difference. The two most important parameters that will determine the system’s efficacy are choosing the retained symptom and the reliability coefficient. As a result, both variables must be calculated very carefully. The color difference E between a*, b* two colors concerning its L*, a*, b*, Equation (11) calculates the variable values.

(11) $$\;\Delta E_{ab}\;=\;\sqrt{{\;\Delta L}^{*}+{\;\Delta a}^{*}+{\;\Delta b}^{*}}$$

where

$$\;\Delta L*=\left(L_{i}^{*}-L_{T}^{*}\right),\;\;\Delta a*=\left(a_{i}^{*}-a_{T}^{*}\right)\;and\;\;\Delta b*=\left(b_{i}^{*}-b_{T}^{*}\right)$$

where $L_{i}^{*}$ , $a_{i}^{*}$ and $b_{i}^{*}$ are the 3 input image channels, $L_{T}^{*}$ , $a_{T}^{*}$ and $b_{T}^{*}$ in the LAB color space, are used to display the template image.

**Figure 3:**
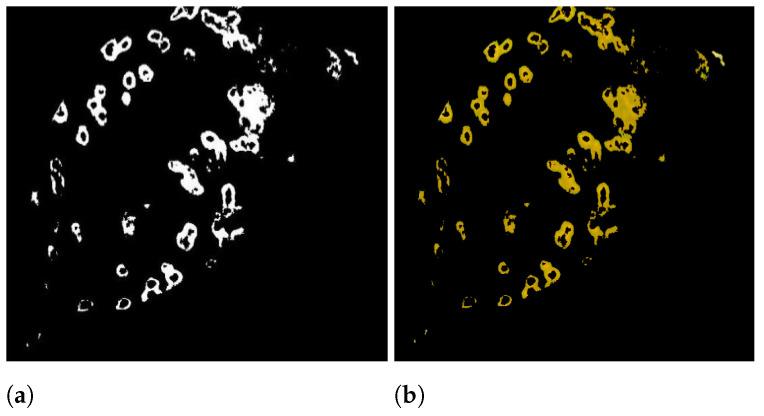
(**a**) Image in binary, (**b**) Segmented image.

Figure 3a displays the final image after applying ΔE segmentation. The disparity in lightness is denoted by the letter ΔL . The difference between red and green colors is Δa , and the difference between the colors yellow and blue is Δb .

When the threshold is applied to the input image $I_{input}$ , $I_{binary}$ is obtained in Equation (12).

(12) $$I_{binary}=\left\{\begin{array}{c} 1\;\;\;\;\;\;\;\;\;\;\;\;\;\;\;\;\;\;\;if\;I_{input}\leq T\;\\ 0\;\;\;\;\;\;\;\;\;\;\;\;\;\;\;\;\;\;\;\;\;\;Otherwise \end{array}\right.$$

where *T* is the measured threshold by Δ E.

#### 3.4.2. Obtaining RGB Image from Binary Image

After the DE algorithm’s acquisition of a binary image. Multiplying the corresponding (one-to-one) elements of the segmented binary image with the input image as equated in Equation (13) yields the colored segmented image.

(13) $$I_{{RGB}_{seg}}=I_{binary}\times \;I_{input}$$

where × is the multiplicative operator, $I_{binary}$ is a binary image on which segmentation is applied, and $I_{input}$ is the original image. After the cross-ponding pixels have been multiplied, $I_{{RGB}_{seg}}$ is the output image.

### 3.5. Feature Extraction

Color seems to be the best descriptor while dealing with plant images that are affected by the disease. Symptoms of diseases are distinguished chromatically. Our proposed technique deals with features based on the colors of input images. These characteristics are obtained from the segmented image’s histogram.

A histogram well and accurately represents the numerical data. It is an estimate of a continuous variable’s probability distribution (quantitative variable). Every channel’s histogram is calculated for feature extraction. Additionally, as shown in Figure 1, the set of features is generated by concatenating features into an array 6.

### 3.6. RGB and HSV Histogram Features

Plant images are captured in a three-channel RGB color space, with each channel containing unique information. The red channel of a picture contains more data, while the blue channel contains less information. The histogram is a graphical representation of images representing the total number of intensity level frequencies [67]. The frequencies of intensities alter the appearance of images so that each pixel’s position is no longer significant. Color histograms as features have rotation invariance due to this property of histograms. The HSV feature gives the illumination invariance induced by different lighting conditions.

RGB features are calculated as in Equation (14)

(14) $$\left\{R,G,B\right\}=\left[\sum_{i=0}^{L}i_{R}\sum_{i=0}^{L}i_{G}\sum_{i=0}^{L}i_{B}\right]$$

where *L* reflects the total number of gray levels, $i_{R},\;i_{G}\;and\;i_{B}$ represents the frequency of the ith gray level of the image’s *R*, *B*, and *G* channels.

**Figure 4:**
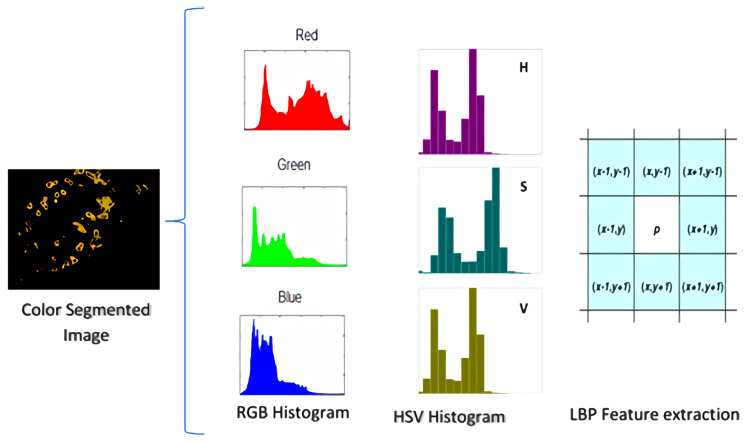
Illustration of HSV histograms, RGB histograms, and LBP feature extraction.

The *R*, G, and *B* image is translated to the HSV color space, and individual channels are concatenated for the HSV histogram features. The concatenation is shown in Figure 4 and is expressed in Equation (15)

(15) $$\left\{H,S,V\right\}=\left[\sum_{i=0}^{L}i_{H}\sum_{i=0}^{L}i_{S}\sum_{i=0}^{L}i_{V}\right]$$

where *L* refers to the total number of gray levels, $i_{H},\;i_{S}\;and\;i_{V}$ represent the value of frequency of *i*-th gray the level of the image’s *H*, *S*, and *V* channels.

Table 3 represents a single RGB image with 255 attributes or an HSV image channel equal to gray levels in images.

The feature sets f1 , f2 , f3 , and f4 display the images’ red, green, blue, and concatenated the RGB histograms. Likewise, for both RGB and HSV attributes, the hue, saturation, value, and concatenated HSV histogram features are f5 , f6 , f7 , and f8 , with a dimension of 765. The feature set f9 with 1536 dimensions is the combined set of RGB and HSV features. The {RGB, HSV} combined collection is generated by putting the HSV histogram features first, then the RGB histogram features.

### 3.7. Local Binary Patterns

The textural identifiers extracted from photographs to construct a features list are referred to as LBP. Moving a frame around the image and evaluating the values of neighboring pixels with the central pixel as a baseline, then defining binary values, creates the LBP feature set. If a neighboring pixel’s value is greater than the center pixel’s value, the value given to the neighboring pixel is 1, otherwise 0. Furthermore, as equated in Equations (16) and (17), the decimal number assigned to the central pixel is determined by the binary of the neighboring pixel.

(16) $$LBP=\sum_{p=0}^{P}v\left(i_{p}-i_{c}\right)2^{P}$$

where $i_{p}$ denotes the current pixel’s value, and $i_{c}$ the value of the central pixel, for LBP calculations, *P* stands for the value of binary numbers and calculates the radius of the window. If *p* is an even number, it refers to the 8th most significant neighbor and has more weight. As shown in Figure 4, when *p* is 0, the neighborhood size is 8 since the *p*-value is the least important.

(17) $$V\left(l\right)=\left\{\begin{array}{c} 1\;\;\;\;\;\;\;\;\;\;if\;\;l\geq T\\ \;0\;\;\;\;\;\;\;\;\;Otherwise \end{array}\right.$$

where *T* represents the window’s threshold or the window’s central pixel’s shifted value over every pixel. V(l) is a phase function that determines each pixel’s locally binary value based on the weighted sum of its neighbors’ pixels.

The LBP histograms are computed after the binary function. Each channel’s LBP is 255, and the combined LBP has 768 dimensions, while the concatenated RGB, HSV, and LBP feature set in f14 has 2304 dimensions. To shape an LBP feature set, LBPs are determined for each RGB image channel as equated in Equation (18).

(18) $$LBP=\left[{\left(\sum_{p=0}^{P}v\left(i_{p}-i_{c}\right)2^{P}\right)}^{R}\;\;{\left(\sum_{p=0}^{P}v\left(i_{p}-i_{c}\right)2^{P}\right)}^{G}\;\;{\left(\sum_{p=0}^{P}v\left(i_{p}-i_{c}\right)2^{P}\right)}^{B}\right]$$

### 3.8. Classification

To train classifiers, the extracted features are used. The features of guava fruit images train the classifiers. The proposed system is trained using the K-fold cross-validation technique. K=5 has been selected as the value. Different classifiers are used to train the same feature set, such as KNN, SVM, Complex tree, Boosted tree, and Bagged tree, to see which one performs the best. A total dataset of 393 guava images are used for detection and classification, with 77, 83, 76, 70, and 87 from Canker, Mummification, Dot, and Rust images, respectively, and 87 from healthy plant images.

For the classifier (SVM), we used the cubic kernel. For a fine KNN classifier with one neighbor and the distance weight in Euclidean distance, metrics are set to 1. The number of learners is 200, where the learner form of the decision tree is used with the boosted tree ensemble process. With subspace dimension 1, the learning rate is set to 0.1. Boosted algorithms make use of the shallow tree, which takes less time and memory. Consequently, it provides a more approximate solution. A related ensemble approach to Boosted tree ensemble configuration is Learner 200, learner rate set to 0.1, bagged tree and learner sort decision tree, subspace dimension 1. The Bagged approach penetrates deeply into a tree and needs more memory and preparation time, resulting in slow prediction. The generalized error is also estimated by the bagged tree ensemble classifier without the need for additional cross-validation. The learner model of decision tree with the Gini Diversity Index split criterion; is the maximum number of splits is 100 when using the Complex tree.

## 4. Results and Discussions

### 4.1. Dataset

With the support of a domain expert, we built a dataset of guava images to illustrate our proposed framework. A high-resolution digital single-lens reflex (DSLR) camera, Canon EOS 1300D (Canon Inc., Tokyo, Japan) with a 22.3×14.9 mm Complementary Metal Oxide Semiconductor (CMOS) 18 MP sensor with a pixel density of 5.43 MP/cm^2^, was used to capture images. The segmented region’s timbral features are extracted after the color histogram features have been checked for accuracy. Without a standard field of view, the photographs are taken in a variety of lighting conditions. The different groups of the dataset are shown in Table 4. There are a total of 393 images in the dataset: 306 images of diseased guava plants and 87 images of healthy (normal) guava plants. There are four subcategories of infected plant images: Canker (77), Mummification (83), Dot (76), and Rust (70) images. The size of each image is 6000×4000 dimensions with 300 dpi resolution. The dataset used for this study is publicly available at [68].

**Figure 5:**
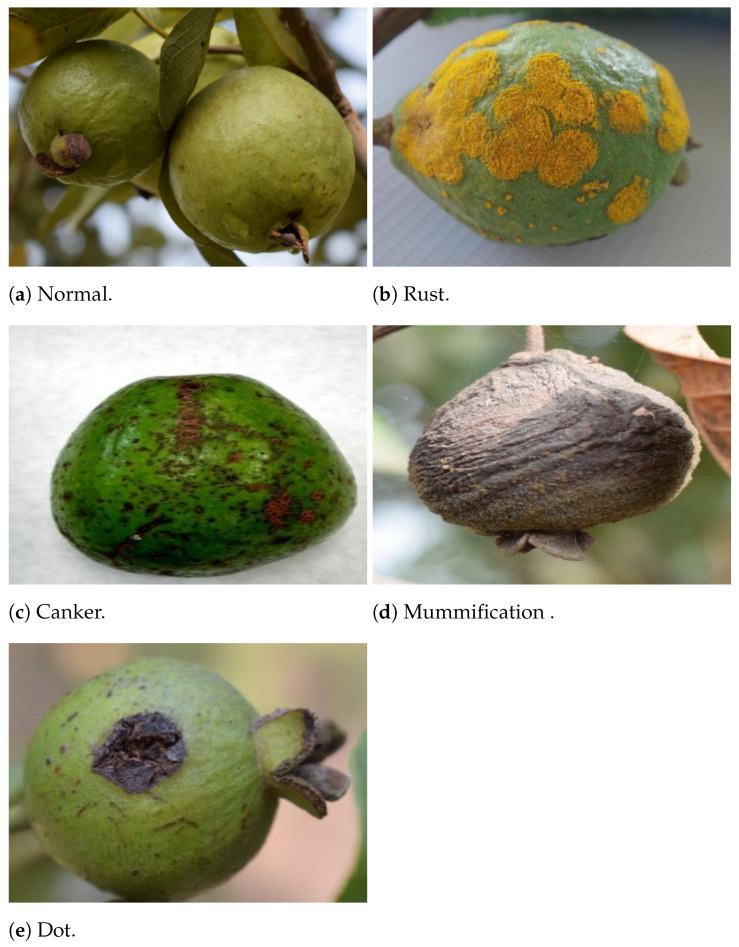
Sample images labeled with each target class (Guava disease).

Images sample of Figure 5a are normal, and others in Figure 5b–e are infected from Rust, Canker, Mummification, and Dot, respectively.

### 4.2. Performance Evaluation

Sensitivity TPR (True Positive Rate), specificity TNR (True Negative Rate), and accuracy are the performance indicators that are used to compare the classifier output as presented in Equations (19)–(21).

(19) $$Sensitivity=\mathrm{TPR}=\frac{\mathrm{TP}}{\mathrm{FN}+\mathrm{TP}}$$

(20) $$Specificity=\mathrm{TNR}=\frac{\mathrm{TN}}{\mathrm{TN}+\mathrm{FP}}$$

(21) $$Accuracy=\frac{\mathrm{TP}+\mathrm{TN}}{\mathrm{TP}+\mathrm{FP}+\mathrm{FN}+\mathrm{TN}}$$

where TP is True Positive, TN is True Negative, FP is False Positive, and FN is False Negative.

### 4.3. Results

The classifier divides images into normal (N) and diseased (A) categories of classification based on images (P). Furthermore, the classification technique identifies images into different diseases at the disease level (e.g., Can, Mum, Dot, Rus, and Nor refer to Canker, Mummification, Dot, Rust diseases, and normal images, respectively).

After feature extraction, the classifiers are trained. Table 5, Table 6, Table 7 and Table 8 show the image-level classification results on testing images. Ranks obtained using the Kruskal-Wallis Test based on the mean accuracy and the performance deviating from the mean of one classifier to the mean of other classifiers using hybrid features are presented in Table 9.

Scale, illumination, and rotation invariance can result from this inconsistency in image acquisition. The image-level accuracy on the LBP features is shown in Table 7. The Fine KNN, Complex Tree, and Bagged Tree ensemble methods are more effective with LBP features. On both color and texture descriptors, the boosted tree ensemble approach performs terribly. Bagged tree and Complex tree outperform other classifiers on color (RGB, HSV) and texture (LBP) attributes.

The image-level classification performance for each separate RGB channel and combined RGB is shown in Table 5. The Bagged tree and Complex tree produce better results on RGB color features, as shown by the results. Cubic SVM performs poorly on the R channel histogram features as compared to Fine KNN. The image-level accuracy for the H, S, and V individual channel features and combined HSV features is shown in Table 6. Fine KNN, Bagged tree, and Complex tree performs well on the HSV color features.

**Figure 6:**
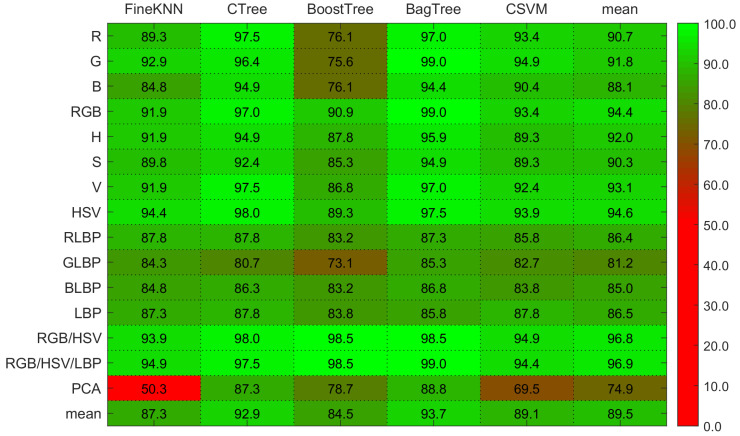
Illustration of heat-maps accuracy comparison using RGB histogram, HSV histogram, LBP and hybrid feature vectors for Guava disease recognition.

**Figure 7:**
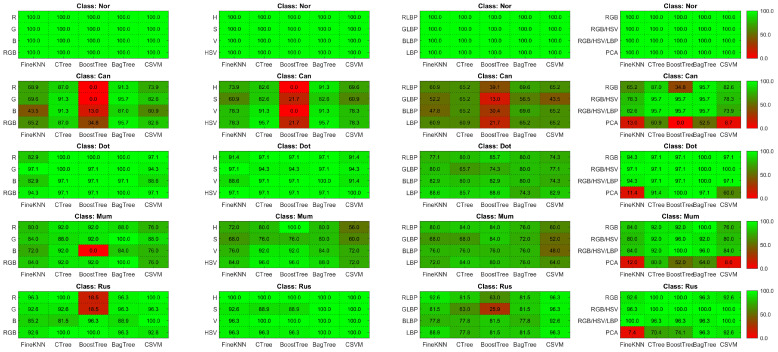
Illustration of heat-maps TPR comparison using RGB histogram, HSV histogram, LBP and hybrid feature vectors for Guava disease recognition.

On RGB color properties, Figure 6 shows that the Bagged tree ensemble approach outperforms the Boosted tree ensemble approach, and the analysis shows that the Bagged tree ensemble approach outperforms the Boosted tree ensemble approach. The disease-level accuracy on the H, S, and V individual channels and combined HSV is also shown in Figure 6 and Figure 7. Complex tree outperforms Fine KNN and Cubic SVM when it comes to HSV color features. The accuracy of disease level on the LBP features is also represented in the same heat-maps. With LBP features, the Complex Tree is more accurate.

Multiple disease class labels are given to training classifiers to achieve disease recognition (Can, Mum, Dot, Rus, and Nor). The Fine KNN, Complex Tree, SVM, Booted tree, and Bagged tree classifiers are trained on similar selected features. The outcomes of classification on test data images are shown in Figure 6 and Figure 7. The Bagged tree and Complex Tree classifiers outperform the Fine KNN, Cubic SVM, and Booted Tree classifiers at the disease level, identical to the image-level classification results.

The classification results in Table 5, Table 6, Table 7 and Table 8 demonstrate the significance of color features. The outcomes of disease-level success are compared. With the same parameters and classifiers, the combination of features reveals the advantages of color and textural features. Table 8 compares and contrasts the performance of these hybrid features. All the experiments and results represent that color, and textural attributes are essential in identifying plant diseases. Combining these characteristics is also useful for detecting and locating diseased areas in plants.

The Bagged tree ensemble approach had a 99% success rate on the RGB features in disease-level performance, while on the HSV and LBP features, the Complex tree achieved 98% and 87.3% accuracy, accordingly. While the other classifiers, such as Boosted tree and Cubic SVM, perform well on RGB and HSV features, they do not perform well on the LBP features. The bagged tree ensemble method performed better than the boosted tree.

Finally, Figure 7 shows the results for Guava disease recognition. The Boosted tree has achieved the best accuracy of 99% on a set of RGB, HSV, and LBP features while outperforming other classification methods due to its underfitting nature.

Overall, we can observe that using histogram features reduced the overfitting for the Rus target class. However, the normal class, in this case, is still overfitted with 100% accuracy. Similarly, TPR was reduced to 87% with the Can target class. The HSV histogram feature vectors further tackle this entire phenomena with 98.5% accuracy using cubic SVM.

Similarly, LBP feature vectors show the worst accuracy for the Can class using all classifiers with less than 70% accuracy. As opposed to this, the GLBP channel and Complex Tree tend to increase the TPR rate of 100%. The observation with LBP feature vectors was underfitting.

Fusing both histogram and LBP feature vectors with optimal parameter setting overcome the underfitting and overfitting problem with 100% TPR rate using Fine KNN, Boosted Tree and Bagged Tree. However, the remaining two classifiers are bound to a 99.1% TPR rate, respectively.

## 5. Conclusions

Guava diseases have become a significant problem because they could be responsible for a significant drop in agricultural products’ quality and quantity, thus hindering sustainability in agriculture. Improper diagnosis can lead to substantial financial losses for farmers. In this paper, we presented a framework for recognizing and classifying diseases in guava plants. For evaluation, we used a high-resolution guava leaf and fruit dataset. We used Δ E segmentation to obtain color histogram RGB, HSV, and textural LBP descriptors. We used advanced classifiers such as Fine KNN, Cubic SVM, Complex tree, Boosted tree, and Bagged tree ensemble for image-level and disease-level classification. When using RGB and HSV color features, the Bagged tree ensemble classifier outperformed other classifiers. Furthermore, the Complex tree classifier outperformed other classifiers when using textural (LBP) features. In the case of the Bagged tree ensemble classifier, color features provided the best disease-level discrimination. Overall, the classification accuracy is 99%.

In the future, we intend to extend this work by employing deep learning methods to extract features automatically instead of using handcrafted features.

## Figures and Tables

**Table 1:** Summary of existing methodologies on plant disease recognition.

Ref	Plants	Method	Segmentation	Feature Extraction	Classification Technique
[42]	Weeds/corn	Using color features, determine whether the apples are ripe enough to harvest.	color-based background subtraction	Energy features	BPNN
[53]	Redgrape fruit	Suppurating and usual images, as well as disease symptoms	-	Textural features	Spectral information
[54]	Citrus	Data are obtained using a citrus UAV, and images of plants are analyzed using sensors	-	Regression analysis	Stepwise SVM, LDA and QDA
[13]	Beats	Leaf blight spot, leaf rust, and powdery mildew are classified.	-	Nine spectral vegetation	SVM
[40]	Multiple plants	PSO feature selection which is kernel-based, is used for optimal feature selection and leaf classification.	Region of Interest	GLCM + LBP	(FRVM)
[34]	Orange fruit	By calculating gray level co-occurrence matrix (GLCM), texture and gray level features of defect area are extracted, and Probabilistic Neural Networks (RBPNN) is used for classification	Hue and Saturation histograms	GLCM	RBPNN
[14]	Citrus	For classification of disease textural features and color histogram were used.	Delta E	RGB, HSV histogram features + LBP	KNN and SVM
[55]	Multiple plants	Plant leaf disease using KNN classifier	color Segmentation	Textural features	KNsVMN
[56]	Citrus plants	Citrus diseases detection using machine-learning feature selection, extraction and classification.	Weighted Segmentation	Textural + color + Geometric features	M-SVM
[57]	Peach tree	Humboldtian diagnosis of peach tree using random forest	-	Meteorological indices and soil and tissue tests	Random Forest
[58]	Banana plants	Banana leaf diseases using enhanced Gabor feature descriptor	-	Gabor filter and 2D log Gabor filter descriptor	KNN
[59]	Mango plants	Disease of mango leaves detection through ANN and Hybrid Metaheuristic descriptor	Binary Segmentation	Textural+ Statistical	ANN
[60]	Apple tree	Used Brightness-preserving dynamic fuzzy histogram equalization	Histogram Equalization	Automatic feature extraction	KNN
[61]	Cucumber plant	Feature fusion and selection techniques for cucumber diseases detection	-	Probability distribution-based entropy	Multiple Classifiers
[62]	Cassava leaves	Cassava mosaic disease recognition using a deep residual convolution neural network (DRNN) with distinct block processing	Distinct block processing	-	DRNN
[63]	Apple leaves	MASK RCNN to detect infected regions, CNN for feature extraction and Kapur’s entropy along multiclass SVM for feature selection	Mask RNN	Kapur’s entropy with multiclass SVM	Ensemble subspace discriminant analysis

**Table 2:** Critics (advantages and drawbacks) of the related works.

Ref	Advantages	Drawbacks
[42]	Wavelet decomposition using different color textures to obtain color bands	Ignored image foregrounds
[53]	Considered Spectral information	Missing statistical features
[54]	Different RGB ranges (R = 900 nm, G = 690 nm and B = 560 nm) used to extraction different color intensities.	Lack k-fold cross validaiton
[13]	Used adaptive template matching for disease development observation	The under-classification problem happened mainly in limited lighting conditions.
[40]	Optimal feature selection using PSO	Only considered ROI
[34]	Used Radial Basis Probabilistic Neural Networks	Lack k-fold cross validaiton
[14]	Combination of ML and Computer-Vision-based approaches	Lack of deep learning-based approaches
[55]	Clustered the corresponding diseases based on color and texture	Lack of deep learning-based approaches
[56]	Combination of ML and Computer-Vision-based approaches	Lack of deep learning-based approaches
[57]	Analyzed Soil conditions	Experiments performed on a small dataset
[58]	Used Gabor filter and 2D log Gabor filter	Lack of deep learning-based approaches
[59]	Extract both Textural + Statistical feature vectors	Adopted Binary Segmentation
[60]	Used Brightness-preserving dynamic fuzzy histogram equalization	Lack of deep learning-based approaches
[61]	Used data augmentation with different angles rotations.	Lack of deep learning-based approaches
[63]	Adopted Kapur’s entropy with multiclass SVM	Lack of handcrafted feature vectors

**Table 3:** Feature sets with dimensions.

Sr #	Feature Sets	Dimensions
1	{H}	255
2	{S}	255
3	{V}	255
4	{HSV}	768
5	{R}	255
6	{G}	255
7	{B}	255
8	{RGB}	768
9	{LBP(R)}	255
10	{LBP(G)}	255
11	{LBP(B)}	255
12	{RGB HSV}	1536
13	PCA{RGB HSV LBP}	195
14	{RGB HSV LBP}	2304
15	{LBP(R) LBP(G) LBP(B)}	768

**Table 4:** Distribution of classes in the dataset.

Description	No of Images
Normal	87
Rust	70
Canker	77
Mummification	83
Dot	76
Total	393

**Table 5:** Results obtained with different classifiers using RGB histogram features for image-level classification.

Channel	R			G			B			RGB		
**Measure**	**TPR**	**TNR**	**ACC**	**TPR**	**TNR**	**ACC**	**TPR**	**TNR**	**ACC**	**TPR**	**TNR**	**ACC**
**Class**	**P**	**N**	**%**	**P**	**N**	**%**	**P**	**N**	**%**	**P**	**N**	**%**
Fine KNN	99.1%	100%	99.5%	100%	100%	100%	100%	100%	100%	100%	100%	100%
Cubic SVM	94.5%	100%	97%	97.3%	100%	98.5%	99.1%	100%	99.5%	99.1%	100%	99.5%
Boosted Tree	100%	100%	100%	100%	100%	100%	100%	0%	55.8%	100%	0%	55.8%
Bagged Tree	100%	100%	100%	100%	100%	100%	100%	100%	100%	100%	100%	100%
Complex Tree	99.1%	100%	99.5%	100%	100%	100%	99.1%	100%	99.5%	99.1%	100%	99.5%

**Table 6:** Combined results obtained using HSV histogram features for image-level classification.

Channel	H			S			V			HSV		
**Measure**	**TPR**	**TNR**	**ACC**	**TPR**	**TNR**	**ACC**	**TPR**	**TNR**	**ACC**	**TPR**	**TNR**	**ACC**
**Class**	**P**	**N**		**P**	**N**		**P**	**N**		**P**	**N**	
Fine KNN	100%	100%	100%	100%	100%	100%	100%	100%	100%	100%	100%	100%
Cubic SVM	97.3%	100%	98.5%	96.4%	100%	98%	97.3%	100%	98.5%	94.5%	100%	97%
Boosted Tree	100%	0%	55.8%	98.2%	100%	99%	99.1%	100%	99.5%	100%	0%	55.8%
Bagged Tree	100%	100%	100%	98.2%	100%	99%	100%	100%	100%	100%	100%	100%
Complex Tree	98.2%	100%	99%	98.2%	100%	99%	100%	100%	100%	98.2%	100%	99%

**Table 7:** Results obtained with different classifiers using LBP features for image-level classification.

Features	RLBP			GLBP			BLBP			LBP		
**Measure**	**TPR**	**TNR**	**ACC**	**TPR**	**TNR**	**ACC**	**TPR**	**TNR**	**ACC**	**TPR**	**TNR**	**ACC**
**Class**	**P**	**N**		**P**	**N**		**P**	**N**		**P**	**N**	
Fine KNN	100%	100%	100%	100%	100%	100%	98.2%	100%	99%	100%	100%	100%
Cubic SVM	96.4%	100%	98%	91.8%	100%	95.4%	95.5%	19.5%	61.9%	100%	100%	100%
Boosted Tree	100%	0%	55.8%	100%	0%	55.8%	100%	0%	55.8%	100%	0%	55.8%
Bagged Tree	99.1%	100%	99.5%	98.2%	100%	99%	99.1%	100%	99.5%	99.1%	100%	99.5%
Complex Tree	99.1%	100%	99.5%	98.2%	100%	99%	98.2%	100%	99%	99.1%	100%	99.5%

**Table 8:** Results obtained with different classifiers using hybrid features for image-level classification.

Features	{RGB, HSV}			{RGB, HSV, LBP}			PCA{RGB, HSV, LBP}		
**Measure**	**TPR**	**TNR**	**ACC**	**TPR**	**TNR**	**ACC**	**TPR**	**TNR**	**ACC**
**Class**	**P**	**N**	**%**	**P**	**N**	**%**	**P**	**N**	**%**
Fine KNN	100%	100%	100%	100%	100%	100%	15.5%	100%	52.8%
Cubic SVM	99.1%	100%	99.5%	100%	100%	100%	59.1%	100%	72.5%
Boosted Tree	100%	0%	55.8%	100%	0%	55.8%	100%	100%	100%
Bagged Tree	100%	100%	100%	99.1%	100%	99.5%	100%	100%	100%
Complex Tree	99.1%	100%	99.5%	99.1%	100%	99.5%	97.3%	100%	98.5%

**Table 9:** Ranks obtained using the Kruskal-Wallis Test based on the mean accuracy and the performance deviating from the mean of one classifier to the mean of other classifiers using hybrid features.

	{RGB, HSV}		{RGB, HSV, LBP}		CA{RGB, HSV, LBP}	
Classifier	Rank	Z	Rank	Z	Rank	Z
Bagged Tree	4.5	1.06	2.5	−0.35	4.5	1.06
Boosted Tree	1.0	−1.41	1.0	−1.41	4.5	1.06
Complex Tree	2.5	−0.35	2.5	−0.35	3.0	0.00
Cubic SVM	2.5	−0.35	4.5	1.06	2.0	−0.71
Fine KNN	4.5	1.06	4.5	1.06	1.0	−1.41

## Data Availability

The dataset used for this study is publicly available at [68].
