# Correction: Almadhor et al. AI-Driven Framework for Recognition of Guava Plant Diseases through Machine Learning from DSLR Camera Sensor Based High Resolution Imagery. *Sensors* 2021, *21*, 3830

**DOI:** 10.3390/s24248210

**Published:** 2024-12-23

**Authors:** Ahmad Almadhor, Hafiz Tayyab Rauf, Muhammad Ikram Ullah Lali, Robertas Damaševičius, Bader Alouffi, Abdullah Alharbi

**Affiliations:** 1 Department of Computer Engineering, Networks Jouf University, Sakaka 72388, Saudi Arabia; aaalmadhor@ju.edu.sa; 2 Independent Researcher, Bradford BD8 0HS, UK; 3 Department of Information Sciences, University of Education Lahore, Lahore 41000, Pakistan; m.i.lali@ue.edu.pk; 4 Faculty of Applied Mathematics, Silesian University of Technology, 44-100 Gliwice, Poland; robertas.damasevicius@polsl.pl; 5 Department of Computer Science, College of Computers and Information Technology, Taif University, P.O.Box 11099, Taif 21944, Saudi Arabia; balouffi@tu.edu.sa; 6 Department of Information Technology, College of Computers and Information Technology, Taif University, P.O. Box 11099, Taif 21944, Saudi Arabia; amharbi@tu.edu.sa

**Affiliation Correction**

In the original publication [1], the second author’s Affiliation 2 was wrong. The correct Affiliation 2 appears below:

Independent Researcher, Bradford BD8 0HS, UK

The authors state that the scientific conclusions are unaffected. This correction was approved by the Academic Editor. The original publication has also been updated.
